# Combined CCNE1 high‐level amplification and overexpression is associated with unfavourable outcome in tubo‐ovarian high‐grade serous carcinoma

**DOI:** 10.1002/cjp2.168

**Published:** 2020-05-11

**Authors:** Angela MY Chan, Emeka Enwere, John B McIntyre, Holly Wilson, Chidera Nwaroh, Nicholas Wiebe, Young Ou, Shuhong Liu, Katharina Wiedemeyer, Peter F Rambau, Xin Grevers, Donald G Morris, Paola Neri, C Blake Gilks, Frank Visser, Nhu Le, Li Luo, Linda S Cook, Martin Köbel

**Affiliations:** ^1^ Precision Oncology Hub, Department of Oncology University of Calgary, Tom Baker Cancer Centre Calgary AB Canada; ^2^ Department of Pathology and Laboratory Medicine University of Calgary, Foothills Medical Center Calgary AB Canada; ^3^ Department of Pathology Catholic University of Health and Allied Sciences‐Bugando Mwanza Tanzania; ^4^ Department of Cancer Epidemiology and Prevention Research Alberta Health Services‐Cancer Control Alberta Calgary AB Canada; ^5^ Department of Pathology and Laboratory Medicine University of British Columbia Vancouver BC Canada; ^6^ Hotchkiss Brain Institute Molecular Core Facility, Health Research Innovation Centre University of Calgary Calgary AB Canada; ^7^ Cancer Control Research BC Cancer Research Centre Vancouver BC Canada; ^8^ Division of Epidemiology, Biostatistics and Preventive Medicine, Department of Internal Medicine and UNM Comprehensive Cancer Center University of New Mexico Albuquerque NM USA

**Keywords:** ovarian cancer, high grade serous carcinoma, *CCNE1*, cyclin E1, amplification, prognosis, PARP inhibitor

## Abstract

*CCNE1* amplification is a recurrent alteration associated with unfavourable outcome in tubo‐ovarian high‐grade serous carcinoma (HGSC). We aimed to investigate whether immunohistochemistry (IHC) can be used to identify *CCNE1* amplification status and to validate whether *CCNE1* high‐level amplification and overexpression are prognostic in HGSC. A testing set of 528 HGSC samples stained with two optimised IHC assays (clones EP126 and HE12) was subjected to digital image analysis and visual scoring. DNA and RNA chromogenic *in situ* hybridisation for *CCNE1* were performed. IHC cut‐off was determined by receiver operating characteristics (ROC). Survival analyses (endpoint ovarian cancer specific survival) were performed and validated in an independent validation set of 764 HGSC. Finally, combined amplification/expression status was evaluated in cases with complete data (*n* = 1114). *CCNE1* high‐level amplification was present in 11.2% of patients in the testing set and 10.2% in the combined cohort. The optimal cut‐off for IHC to predict *CCNE1* high‐level amplification was 60% positive tumour cells with at least 5% strong staining cells (sensitivity 81.6%, specificity 77.4%). *CCNE1* high‐level amplification and overexpression were associated with survival in the testing and validation set. Combined *CCNE1* high‐level amplification and overexpression was present in 8.3% of patients, mutually exclusive to germline *BRCA1/2* mutation and significantly associated with a higher risk of death in multivariate analysis adjusted for age, stage and cohort (hazard ratio = 1.78, 95 CI% 1.38–2.26, *p* < 0.0001). *CCNE1* high‐level amplification combined with overexpression identifies patients with a sufficiently poor prognosis that treatment alternatives are urgently needed. Given that this combination is mutually exclusive to *BRCA1/2* germline mutations, a predictive marker for PARP inhibition, *CCNE1* high‐level amplification combined with overexpression may serve as a negative predictive test for sensitivity to PARP inhibitors.

## Introduction

Over the past decades, the 5‐year survival rate for patients with tubo‐ovarian high‐grade serous carcinomas (HGSC) has slightly improved from 27 to 35% to reach 40% in most Western countries [1]. Molecularly, HGSC are characterised by the ubiquitous presence of inactivating *TP53* mutations and copy number alterations [2, 3, 4]. Seven distinct copy number signatures have been described in HGSC, and some are a consequence of homologous recombination repair deficiency (HRD) [5]. The prototypical HRD alteration is germline or somatic *BRCA1/2* mutation, which occurs in 23% of patients with HGSC [6].

Another mechanism, fold‐back inversions, causes localised amplifications such as *CCNE1* amplification, which occurs in 22% of patients with HGSC [7, 8]. *CCNE1* amplifications are an early event because of their presence in a subset of HGSC precursor, serous tubal intraepithelial carcinoma [9, 10]. *CCNE1* gains and *BRCA1/2* germline mutations are inversely correlated, which becomes mutually exclusive with high‐level (>8 copies) *CCNE1* amplification [7, 11]. *CCNE1* amplified HGSC often show increased ploidy by whole genome duplication due to failed cytokinesis [12]. *CCNE1* amplification has been consistently associated with unfavourable survival in HGSC patients and linked to chemo‐resistance and primary treatment failure [7, 13, 14, 15]. *In vitro* work has demonstrated that *CCNE1* amplified tumours are sensitive to CDK2 or proteasome inhibition [11, 12]. Therefore, *CCNE1* amplification status might inform stratification of patients with HGSC to targeted therapies.

Despite extensive study of *CCNE1*, no validated assay is available to examine *CCNE1* amplification for clinical trial inclusion. Immmunohistochemistry (IHC) could potentially serve as a screening test analogous to the clinical test algorithm for *ERBB2* amplification in breast cancer [16]. However, previous studies showed only a moderate correlation of *CCNE1* amplification with protein expression in HGSC [14, 17]. Based on our recent encouraging experience with p53, where optimised IHC provides a clinically useful prediction of the *TP53* mutation status, we hypothesised that IHC could be optimised to serve as a useful screening test for *CCNE1* amplified cases [4]. Furthermore, the prognostic value of CCNE1 protein expression is not well defined. Previous studies were heterogeneous regarding the number of positive cases (ranging from 31 to 68%), use of different antibodies, cut‐offs and inclusion of histotypes other than HGSC [14, 18, 19, 20, 21, 22, 23]. One study (restricted to HGSC, *n* = 262) suggested that the combination of *CCNE1* amplification and protein overexpression is associated with an unfavourable outcome [14].

The aims of this study were to test whether IHC is sufficiently accurate to identify *CCNE1* amplification status and to validate whether *CCNE1* high‐level amplification and CCNE1 protein overexpression are prognostic in HGSC using large training and validation sets in accordance with the National Academy of Medicine recommendations for translational biomarker studies, as well as REMARK [24, 25]. A secondary aim was to explore the survival association of combined *CCNE1* high‐level amplification and overexpression.

## Patients and methods

### Patients and samples

We assembled a small optimisation cohort (*n* = 48) and separate testing and validation sets. Each case was represented on tissue microarrays (TMAs) by at least two cores of 0.6mm punches [26, 27]. The testing set is from the Ovarian Cancer in Alberta and British Columbia (OVAL‐BC) study, which recruited incident cases of ovarian carcinoma from provincial cancer registries of two Canadian provinces between 2001–2012(BC) and 2005–2011(AB) [28]. The validation set is from the Canadian Ovarian Experimental Unified Resource (COEUR), which collected over 2000 ovarian carcinoma cases from 12 Canadian centres between 2010 and 2017 [29]. Both sets were subjected to histopathological review with the integration of immunohistochemical markers (WT1/p53) to confirm HGSC [26, 30]. After removing duplicate cases, 528 HGSC were available for the testing set and 764 for the validation set. Ethics/IRB approval was given by the Health Research Ethics Board of Alberta (HREBA.CC‐18‐0309).

### *CCNE1* DNA/RNA chromogenic *in situ* hybridisation, NanoString and digital PCR

An in‐house chromogenic *in situ* hybridisation (CISH) protocol using a commercial DIG‐labelled *CCNE1* DNA probe (Empire Genomics, Buffalo, NY, USA) and RNA probe (ACDBio, Newark, CA, USA) was developed. Four micrometre sections were cut from TMA blocks, de‐paraffinised, and pretreated with proteinase K, citrate‐based antigen retrieval buffer and pepsin. CISH, NanoString and Digital PCR for *CCNE1* was performed on a small optimisation cohort. Details are provided in Supplementary materials and methods.

### Inducible cell line control

Lentivirus was used to stably transduce K562 cells (ATCC, CCL‐243, Old Town Manassas, VA, USA) with the *CCNE1* gene under the Tet‐On system allowing inducible expression of CCNE1 by addition of varying amounts of Doxycycline. Details of packaging are provided in Supplementary materials and methods. With the addition of Doxycycline, the TRE3G promoter driving CCNE1 expression packaged on a second lentivirus will then respond to the Doxycycline bound Tet activator to induce expression of CCNE1 and mCherry. Hence, cells with successful transduction of both lentiviruses will show both GFP and mCherry expression and appear yellow under a fluorescent microscope. Doubly transduced cells were flow‐sorted by the medium intensity in bulk. Use of the EF1a constitutively active promotor is preferred due to its being less susceptible to silencing; therefore, CCNE1 expression can be tightly controlled by the amount of Doxycycline added.

### Immunohistochemistry

Four micrometre sections were cut from TMA blocks, deparaffinised and rehydrated. Heat‐induced epitope retrieval was performed on‐board the DAKO Omnis platform followed by incubation of CCNE1 antibodies (Supplementary materials and methods) at room temperature, and the Dako EnVision FLEX (Dako, Denmark). The reaction was visualised using 3,3‐diaminobenzidine tetrahydrochloride for 10 min, then haematoxylin as counterstain.

### Digital image analysis

Automated image acquisition was performed using an Aperio Scanscope XT (Aperio Inc., Vista, CA, USA). Images were then analysed using the Indica Labs HALO programme version 2.0.1145.14. For each patient TMA spot a tumour‐specific inclusion area was manually annotated with the aid of a serial section stained with pan‐cytokeratin. Unusable areas such as folded or necrotic tissue were manually cropped. TMA cores were included in the analysis if: (1) at least half of the image was usable and (2) >200 cells per TMA core were present. The analysis algorithm allowed the data acquisition of average pixel intensity in the annotated area as well as absent, weak, moderate and strong intensity gauged by a pathologist (MK). All images were processed using the same thresholds and all subsequent image manipulations involved only image information from the inclusion area. Optical density was calculated by the image analysis software using log10 (*white in/x*), where *white in* is 240 and *x* is pixel for the stain after colour deconvolution.

### Visual scoring

An example image library was created from cases with image analysis assessment (Supplementary materials and methods). Distribution % was assessed in a 10–20% tier categories blinded to outcome data. A second observer scored subsets for inter‐observer reproducibility assessment.

### Statistical analysis

Maximum values were used for cases represented by more than one core and discordant values. IHC cut‐off was determined by receiver operating characteristics (ROC). Inter‐observer reproducibility was estimated using kappa statistics. Associations of *CCNE1* amplification and expression with clinicopathological variables were examined using the chi^2^ test for binary and categorical variables. The log‐rank tested Kaplan–Meier plots for differences. The primary end‐point, ovarian carcinoma‐specific survival, was defined as the time interval between the date of histological diagnosis and the date and time of death from ovarian cancer. Hazard ratios (HRs) were estimated from multivariable cox regression model adjusted for age, stage, surgical outcome and platinum‐based chemotherapy. The study adheres to REMARK guidelines (Supplementary materials and methods) [25]. Statistical analyses were performed in JMPv14 (SAS, Institute, Cary, North Carolina, USA).

## Results

### *CCNE1* assay development

**Figure 1:**
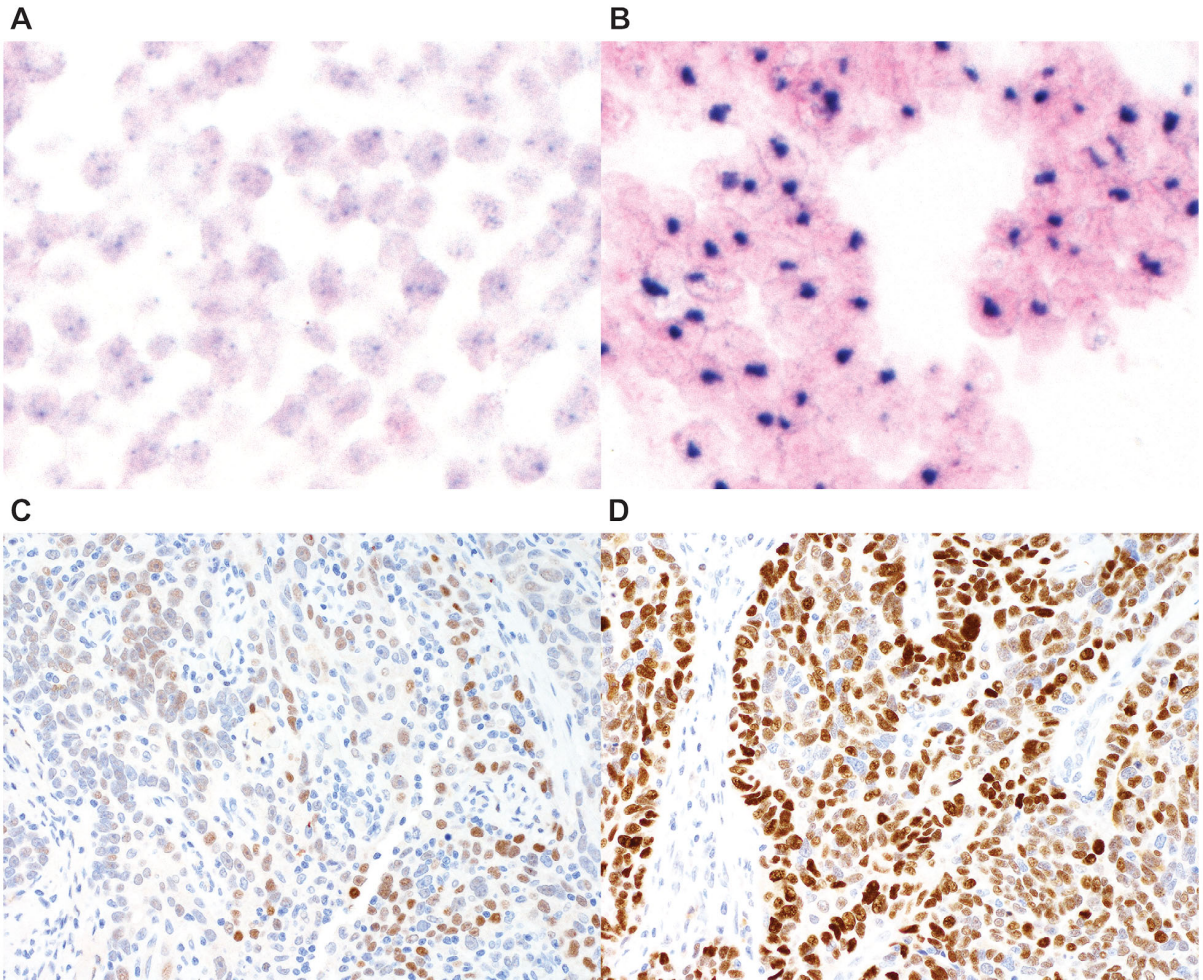
*CCNE1* DNA CISH and IHC. (A) Tubo‐ovarian high‐grade serous carcinoma without amplification (original total magnification ×400). (B) High‐level amplification of *CCNE1* evident by clearly visible nuclear clusters of CISH signal (original total magnification ×400). (C) Tubo‐ovarian high‐grade serous carcinoma with low CCNE1 expression by IHC. (D) Tubo‐ovarian high‐grade serous carcinoma with CCNE1 overexpression (>60% of tumour cells staining with >5% strongly staining).

**Figure 2:**
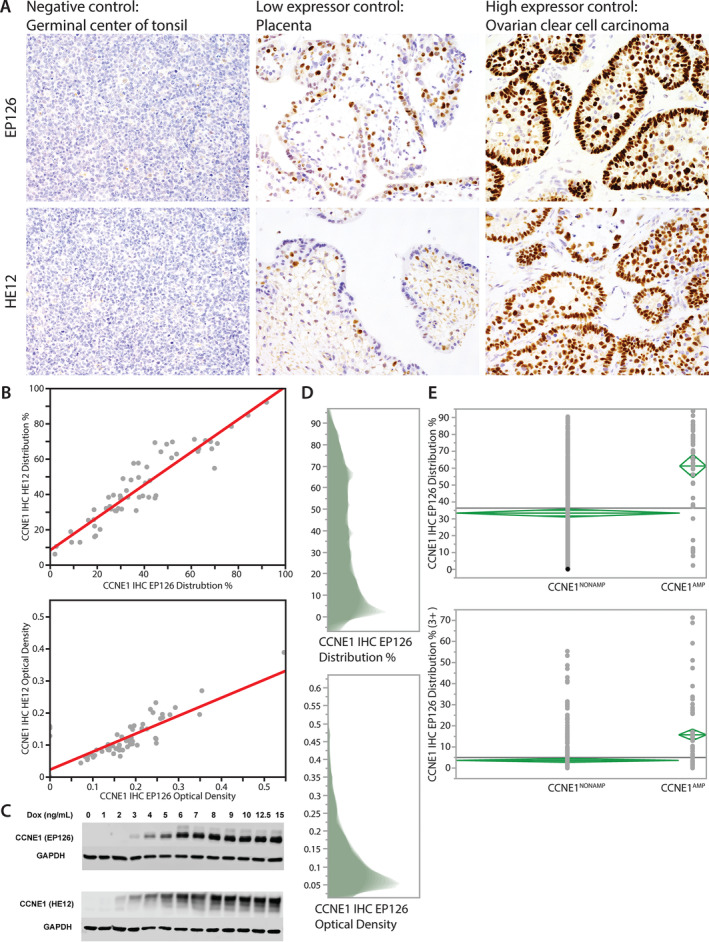
Multi‐step CCNE1 IHC assay standardisation. (A) IHC controls for 2 different IHC assays (clones EP126 and HE12). (B) Image analysis data (% positive cells, optical density) from two different IHC assays. (C) Western blot of inducible cell lines. (D) The distribution of percentage positive tumour cells and optical density of the maximum cores analysed by image analysis of IHC on the testing cohort using clone EP126 assay. (E) Determination of the optimal cut‐off for distribution of percentage positive tumour nuclei by IHC to predict *CCNE1* high level amplification by CISH. Upper panel, all positive tumour cells; lower panel, only strongly staining tumour cells (3+).

Forty‐eight HGSC cases were used to optimise DNA and RNA CISH as well as IHC. *CCNE1* DNA copy number status was assessed using NanoString and digital PCR (Supplementary materials and methods and Figure S1). DNA CISH assay was optimised to detect high‐level amplification of >8 copies of *CCNE1*, as defined by NanoString and digital PCR, by displaying dense signal clusters (Figure 1 and Supplementary material, Table S1). For IHC, 2 commercial antibodies (clone EP126 and clone HE12) showed specific staining in control tissue (Figure 2A). When examined using image analysis, both IHC assays showed an excellent correlation (spearman *R* = 0.91 for % positive cells [maximum across cores] and 0.76 for optical density (maximum across cores, Figure 2B). Specificity of both antibodies was confirmed using the inducible cell lines *via* Western blot and IHC on the cell blocks (Figure 2C and Supplementary material, Figure S2). However, the correlation between IHC and *CCNE1* copy number by NanoString/digital PCR was only moderate for both antibodies (Spearman 0.35 for EP126 and 0.49 for HE12, see Supplementary material, Figure S3). Hence, the optimisation cohort did not identify a clearly superior antibody clone that provided a high degree of correlation with *CCNE1* high‐level amplification, nor did it identify an optimal cut‐off of the IHC assay in predicting high‐level amplification.

### Accuracy of CCNE1 IHC to predict *CCNE1*high‐level amplification in the testing set

Both IHC assays were then applied to the 528 cases testing set. Due to the slightly better signal to noise ratio, we decided to analyse clone EP126 with automated image analysis. The distribution of % positive tumour cells and maximal optical densities are shown in Figure 2D. The right skewed distribution of the continuous data did not suggest a naturally occurring cut‐off in this large testing set. Next we tried to determine the optimal cut‐off for percentage positive tumour nuclei by IHC to predict *CCNE1* high level amplification by CISH. The mean percentage positive tumour cells was significantly higher in *CCNE1* high‐level amplified compared to non‐amplified cases; 61.2% (95% CI 54.4–68.1%) versus 33.3% (95% CI 30.9–35.7%), *p* < 0.0001 (Figure 2E). In addition, the mean number of strongly staining nuclei was 15.7% (95% CI 13.1–18.4%) in high‐level amplified versus 3.6% (95% CI 2.7–4.6%) in non‐amplified cases. The area under the curve (AUC) to predict high‐level amplification was 0.787 and suggested an optimal cut‐off to predict high‐level amplification at 61% (Supplementary material, Figure S4).

We then performed visual scoring using a 6‐tier system at 10–20% increments. These data showed that visual pathologist scoring correlated well with the image analysis data, *r* = 0.896. There was a slight tendency to visually overcall the percentage score in higher staining cases (Supplementary material, Figure S5). The AUC for the 6‐tier interpretation to predict high‐level amplification increased to 0.825 (Supplementary material, Figure S6). ROC analysis yielded a ≥60% cut‐off as the optimal cut‐off to predict high‐level amplification (AUC = 0.771, sensitivity 73.1%, specificity 81.1%; Supplementary material, Table S2). The inter‐observer agreement between two raters using the ≥60% cut off achieved a Cohen's kappa = 0.79 (percentage inter‐rater agreement 91.9%). Discordant cases were reviewed and for subsequent scoring it was decided that a combination of at least 60% positive tumour cells with at least 5% strongly staining cells is considered CCNE1 overexpression (CCNE1^hi^, Figure 1). Using this cut‐off, IHC achieved moderate accuracy for predicting high‐level amplification in the validation set (AUC = 0.812, sensitivity 88.7%, specificity 74.8%; Supplementary material, Table S2), and sensitivity 81.6% and specificity 77.4% in the combined cohort (Supplementary material, Table S3).

### Associations of *CCNE1* high‐level amplification, *CCNE1* RNA CISH and overexpression with survival of HGSC patients in the testing set

Basic clinical characteristics are depicted in Supplementary material, Table S4. *CCNE1* high‐level amplification, mRNA (Supplementary material, Figure S7) and protein expression were assessed in the testing set (Table 1). All IHC were significantly associated with survival in multivariable analysis (Table 2; individual Kaplan–Meier survival curves are shown in Supplementary material, Figure S8). *CCNE1* DNA CISH showed borderline significance and RNA CISH was not significantly associated with survival. There was a moderate correlation between RNA CISH scores and DNA CISH status (*r* = 0.273), mRNA CISH and protein expression (*r* = 0.468).

**Table 1:** Frequency of CCNE1 protein expression by IHC, amplification by CISH and RNA expression by CISH.

CCNE1 assay	Total	Testing set	Validation set
	1292	528	764
*CCNE1* DNA CISH			
Non‐amplified	1000 (89.8%)	412 (88.8%)	588 (90.5%)
High‐level amplification	114 (10.2%)	52 (11.2%)	62 (9.5%)
Missing	178	64	114
CCNE1 IHC (EP126, automated), median of % positive cells (interquartile range)	31.5% (10.9–57.3%)	31.5% (10.9–57.3%)	NA
CCNE1 IHC (EP126, visual)			
<20%	351 (27.2%)	193 (36.6%)	158 (20.7%)
20–39%	394 (30.5%)	130 (24.6%)	264 (34.5%)
40–49%	100 (7.7%)	35 (6.6%)	65 (8.5%)
50–59%	91 (7.1%)	41 (7.8%)	50 (6.5%)
60–79%	220 (17.0%)	69 (13.1%)	151 (19.8%)
80–100%	136 (10.5%)	60 (11.4%)	76 (9.9%)
*CCNE1* RNA CISH			
Absent	47 (10.6%)	47 (10.6%)	NA
Weak	118 (26.7%)	118 (26.7%)	
Moderate	138 (31.1%)	138 (31.1%)	
Strong	140 (31.6%)	140 (3.16%)	
Missing	85	85	

NA, not assessed.

**Table 2:** Multivariable ovarian cancer specific survival analyses of separate assays.

		Testing set	Validation set	Combined cohort
CCNE1 assay	Reference	HR (95% CI, *P* value)	HR (95% CI, *P* value)	HR (95% CI, *P* value)
CCNE1 *DNA CISH*	CCNE1^nonamp^	1.42 (0.99–1.99, *p* = 0.057)	1.67 (1.22–2.23, *p* = 0.0016)	1.47 (1.17–1.84, *p* = 0.0013)
CCNE1 *RNA CISH*	Less than strong expression	1.12 (0.86–1.45, *p* = 0.38)	NA	NA
CCNE1 IHC (EP126, image analysis)	≤60%	1.60 (1.23–2.06, *p* = 0.0005)	NA	NA
CCNE1 IHC (EP126, visual scoring)	CCNE1^lo^	1.47 (1.14–1.88, *p* = 0.0030)	1.27 (1.04–1.54, *p* = 0.019)	1.36 (1.16–1.58, *p* = 0.0001)
CCNE1 IHC (HE12, visual scoring)	CCNE1^lo^	1.47 (1.13–1.88, *p* = 0.0041)	NA	NA

NA, not assessed.

Adjusted for study site, age (continuous), stage (I–IV, unknown), surgical outcome (complete, optimal, suboptimal, unknown), platinum‐based chemotherapy (none, neoadjuvant, adjuvant, unknown).

CCNE1^nonamp^ – negative for *CCNE1* high‐level amplification (≤8 copies by CISH).

CCNE1^lo^ – negative for CCNE1 protein overexpression by IHC with <60% positive tumour cells or <5% strongly staining cells.

### Validation of survival association of *CCNE1* high‐level amplification and overexpression in the validation set

In order to validate significant results in the testing set, *CCNE1* high‐level amplification and protein expression were also assessed in the validation set (Table 1 and Supplementary material, Table S4). Both showed significant survival associations in uni‐ and multivariable analyses (Table 2 and Supplementary material, Figure S8).

### Explorative analysis of combined *CCNE1* high‐level amplification and overexpression

**Figure 3:**
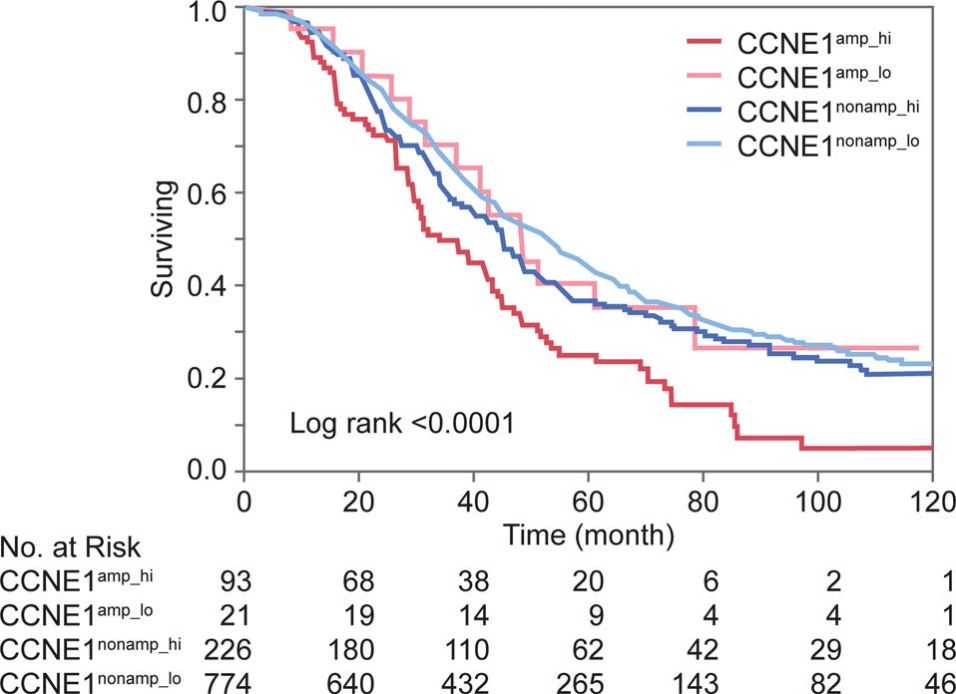
Kaplan–Meier survival analysis of combined *CCNE1* high‐level amplification and overexpression status in tubo‐ovarian high‐grade serous carcinomas. CCNE1^amp^ – *CCNE1* high‐level amplification (>8 copies by CISH); CCNE1^nonamp^ – negative for *CCNE1* high‐level amplification (≤8 copies by CISH); CCNE1^hi^ – CCNE1 protein overexpression by IHC with ≥60% positive tumour cells and ≥5% strongly staining cells; CCNE1^lo^ – negative for CCNE1 protein overexpression by IHC with <60% positive tumour cells or <5% strongly staining cells.

Next, we tested whether a combination of *CCNE1* high‐level amplification and overexpression (CCNE1^amp_hi^) would outperform individual assessments in the testing set. In fact, the HR for the CCNE1^amp_hi^ subgroup compared to reference combination of non‐amplified and low expressing cases (CCNE1^nonamp_lo^) was higher (Table 3) than any separately assessed variable (Table 2). Since this also validated in the validation set, we combined testing and validation sets to generate a combined cohort from here on. Figure 3 shows the unfavourable outcome of the CCNE1^amp_hi^ subgroup with a median survival time of 33.8 months with very poor long term survival. This compares to 51.1 months for CCNE1^nonamp_lo^ and 44.8 months CCNE1^nonamp_hi^ (log rank<0.0001). The HR in multivariable analysis for CCNE1^amp_hi^ compared to reference CCNE1^nonamp_lo^ was 1.84 (95% CI 1.42–2.35, *p* < 0.0001, Table 3). The survival of the CCNE1^amp_hi^ subgroup was also significantly different compared to the other two subgroups (Supplementary material, Table S5). Notably, the survival of the CCNE1^amp_lo^ subgroup was not different from CCNE1^nonamp_lo^ reference. There was a non‐significant trend of a slightly higher risk for the CCNE1^nonamp_hi^ subgroup compared to the CCNE1^nonamp_lo^ reference (Table 3). We estimated the influence of intra‐tumoural heterogeneity on the assay results by assessing the concordance across cores. For CCNE1 IHC (EP126, visual), 1050 of 1292 (92%) of cases were represented by more than one core and 948 of 1050 (90%) showed a concordant result regarding overexpression or not. The concordance for high‐level amplification by CISH was higher across cores (749/756, 99%).

**Table 3:** Multivariable ovarian cancer specific survival analyses of CCNE1 subgroups of HGSC defined by combination of copy number and protein expression status.

		Testing set	Validation set	Combined set
Comparator	Reference	HR (95% CI, *P* value)	HR (95% CI, *P* value)	HR (95% CI, *P* value)
CCNE1^amp_hi^	CCNE1^nonamp_lo^	2.29 (1.51–3.35, *p* = 0.0002)	1.70 (1.22–2.34, *p* = 0.0024)	1.84 (1.43–2.35, *p* <0.0001)
CCNE1^amp_lo^	CCNE1^nonamp_lo^	0.64 (0.28–1.23, *p* = 0.20)	2.21 (0.87–4.62, *p* = 0.90)	0.80 (0.44–1.33, *p* = 0.42)
CCNE1^nonamp_hi^	CCNE1^nonamp_lo^	1.26 (0.91–1.70, *p* = 0.15)	1.19 (0.93–1.50, *p* = 0.16)	1.18 (0.98–1.43, *p* = 0.076)

Adjusted for study site, age (continuous), stage (I–IV, unknown), surgical outcome (complete, optimal, suboptimal, unknown), platinum‐based chemotherapy (none, neoadjuvant, adjuvant, unknown).

HGSC – tubo‐ovarian high‐grade serous carcinoma.

CCNE1^amp^ – *CCNE1* high‐level amplification (>8 copies by CISH).

CCNE1^nonamp^ – negative for *CCNE1* high‐level amplification (≤8 copies by CISH).

CCNE1^hi^ – CCNE1 protein overexpression by IHC with ≥60% positive tumour cells and ≥5% strongly staining cells.

CCNE1^lo^ – negative for CCNE1 protein overexpression by IHC with <60% positive tumour cells or <5% strongly staining cells.

Univariate associations of the four subgroups with clinical parameters and relevant biomarkers are shown in Table 4. Patients with non‐amplified tumours were significantly younger at diagnosis compared to amplified cases. There were no differences regarding stage distribution (*p* = 0.77), residual disease status (*p* = 0.88) and administration of chemotherapy (*p* = 0.56). However, none of the high‐level amplified cases harboured a *BRCA1/2* germline mutation confirming mutual exclusivity. Interestingly, RB1 loss was also mutually exclusive to the CCNE1^amp_hi^ subgroup but was observed in the CCNE1^amp_lo^ subgroup. High frequencies of CDKN2A block expression were seen across the three subgroups with either high‐level amplification or overexpression but not in the CCNE1^nonamp_lo^ subgroup.

**Table 4:** Univariable associations of CCNE1 subgroups of HGSC with clinicopathological parameters and biomarkers.

Variable	Total	CCNE1^amp_hi^	CCNE1^amp_lo^	CCNE1^nonamp_hi^	CCNE1^nonamp_lo^	*P* value
	1114	93 (8.4%)	21 (1.9%)	226 (20.3%)	774 (69.5%)	
Age (mean)	61.4	65.7	67.8	63.2	60.1	<0.0001
CCNE1 mRNA CISH high	134/392 (34.8%)	30/36 (83.3%)	4/13 (30.1%)	35/71 (49.3%)	65/272 (23.9%)	<0.0001
gBRCA1/2 mutation present	43/183 (19.0%)	0/20	NA	4/48 (7.7%)	39/115 (25.3%)	0.0002
RB1 loss by IHC	60/393 (13.2%)	0/38	3/11 (21.4%)	7/69 (9.2%)	50/275 (15.4%)	0.0030
CDKN2A block staining	719/1078 (66.7%)	79/89 (88.8%)	17/21 (81%)	185/221 (83.7%)	438/747 (58.6%)	<0.0001

Numbers represent subtotal because of incomplete data for some markers.

HGSC – tubo‐ovarian high‐grade serous carcinoma.

CCNE1^amp^ – *CCNE1* high‐level amplification (>8 copies by CISH).

CCNE1^nonamp^ – negative for *CCNE1* high‐level amplification (≤8 copies by CISH).

CCNE1^hi^ – CCNE1 protein overexpression by IHC with ≥60% positive tumour cells and ≥5% strongly staining cells.

CCNE1^lo^ – negative for CCNE1 protein overexpression by IHC with <60% positive tumour cells or <5% strongly staining cells.

## Discussion

In this large study of HGSC patients, we validate that separately assessed *CCNE1* high‐level amplification and overexpression are significantly associated with higher risk of ovarian cancer specific death. Against our hypothesis, IHC did not reach sufficient sensitivity to identify all high‐level amplified cases. Using optimised IHC and cut‐off, 18.4% (21/114) of high‐level amplified cases still fell short of overexpression. Lowering the cut‐off, would, however, only yield a marginal increase in sensitivity at a steep cost in specificity. However, we show that the combination of *CCNE1* high‐level amplification and overexpression (CCNE1^amp_hi^) characterises a biologically distinct and particularly aggressive subgroup of HGSC, which has been shown as a trend in a previous smaller study [14].

Our data suggest a biological segregation of the *CCNE1* high‐level amplified cases based on the CCNE1 protein expression status. CCNE1^amp_lo^ cases had a significantly longer survival than CCNE1^amp_hi^. We speculate that concomitant alterations in G1/S transition might be causing this difference. We observed RB1 loss in CCNE1^amp_lo^ but not in CCNE1^amp_hi^ cases. Without RB1, CCNE1 cannot exert its driver function in G1/S transition and CCNE1 transcriptional activity is abrogated as shown by the significantly lower mRNA level in CCNE1^amp_lo^ cases. *CCNE1* high‐level amplified cases are often polyploid due to genome duplication [5, 12]. Hence, the CCNE1^amp_hi^ subgroup might be polyploid making it less likely to sustain genomic RB1 loss [5, 12] while the CCNE1^amp_lo^ subgroup might have a diploid copy number state making it more susceptible to genomic RB1 loss. Future studies should consider CCNE1 in the context of RB1 and other members of the G1/S transition.

One would assume that CCNE1 protein is the decisive factor for CCNE1 function. Both CCNE1 overexpressing subgroups also showed similar proliferative activity as evidenced by high frequency of CDKN2A block expression, a surrogate for high G1/S transition [31]. Despite equivalent protein levels and similar proliferative activity, CCNE1^nonamp_hi^ cases had a longer survival compared to CCNE1^amp_hi^. Normal CCNE1 protein is tightly controlled through a combination of transcriptional and proteasome activity. mRNA levels were significantly higher in CCNE1^amp_hi^ cases indicating transcriptional upregulation caused by copy number abundance. In contrast, protein stabilisation may be the mechanism of CCNE1 overexpression in CCNE1^nonamp_hi^ cases. Aziz *et al* showed that CCNE1^nonamp_hi^ cases have significantly higher USP28 expression, a deubiquitinase that stabilises CCNE1 [14]. Hence, both subgroups have high protein, high proliferation but distinct mechanisms of overexpression and different survival; this raises the possibility of functional differences between stabilised and transcriptionally active CCNE1. An interesting observation is that CCNE1 overexpression never occurred in all tumour cell nuclei in tumour tissue. In the inducible cell line assay, we also observed increased cell death at high (7 ng/ml) doxycycline concentration. This implies that CCNE1 protein overexpression in HGSC cannot be excessive but needs to be regulated to avoid cellular crisis due to uncontrolled cell cycle entry. This also limits the range of CCNE1 expression detectable by IHC. Future studies are required to assess whether HGSC cases defined only by overexpression by IHC (60% positive tumour cells with at least 5% strongly staining cells) without high‐level amplification will require separate treatment. For this purpose, we created an inducible cell line assay, which can be distributed as a control to standardise IHC for clinical trial inclusion. As normal tissue controls we recommend germinal centre of tonsil (negative control), placenta (low expressor positive control) and ovarian clear cell carcinoma (high expressor positive control). In ovarian clear cell carcinoma, CCNE1 overexpression has been also associated with unfavourable prognosis. However, overexpression in this histotype is correlated with low level copy number gain (2.0–2.9 copies) or polysomy but not high‐level amplification as seen in HGSC [32].

We did not see a survival association with *CCNE1* mRNA levels. Quite to the contrary, a recent study of 166 HGSC reported an association of *CCNE1* mRNA expression assessed by qPCR with favourable outcome in multivariate analysis [33]. Future studies are needed to assess whether *CCNE1* mRNA signal is able to detect survival differences but, based on our analysis,DNA copy number and protein levels are superior prognostic indicators.

As a limitation of the study, we had incomplete data for certain analyses. For example, germline *BRCA1/2* mutation status was only available for a subset of patients. CISH assays caused case dropout due to inhomogeneous tissue quality, in comparison to IHC. We validate that high‐level amplification, as defined by easily visible clusters of CISH signals corresponding to >8 copies of *CCNE1* gene by other assays, is mutually exclusive to germline *BRCA1/2* mutations [11]. Although CISH can be applied to TMAs, the resolution of our current CISH assay does not allow reliable quantification of low‐level *CCNE1* gains. The copy number assay presented herein can be applied to TMAs but alternative assays such as digital PCR are also feasible for developing into a clinical test. In keeping with a driver alteration, we observed very little intra‐tumoural heterogeneity for *CCNE1* high‐level amplifications. However, CCNE1 protein expression did show some heterogeneity, which should be more carefully studied in the future.

Our study suggests the importance of a combined assessment of CCNE1 protein expression and *CCNE1* high‐level amplification because they identify biologically distinct subgroups of patients with HGSC; a finding that requires further consortium‐type validation [34]. In particular, the segregation of *CCNE1* high‐level amplified cases by protein status refines the subgroup with the highest risk. Given the confusion around which patients should receive PARP inhibitors [35], this CCNE1^amp_hi^ subgroup is unlikely to respond to PARP inhibitors. This assumption is based on the mutual exclusivity with *BRCA1/2* mutations, a distinct non‐HRD oncogenesis with fold‐back inversion causing focal high‐level amplifications, and poor survival despite being treated with conventional platinum‐taxol chemotherapy. The latter indicates chemotherapy resistance, which is associated with resistance to PARP inhibitors. We therefore propose to test clinical trial material where response to validation PARP inhibitors is known for the CCNE1^amp_hi^ status and hypothesise that this status can serve as a negative predictive test for PARP inhibitors. Alternative treatment options either targeting the mechanism of localised high‐level amplifications (e.g. POLθ [8]) or the downstream effect (e.g. CDK2 inhibitors [36]) should be tested in the CCNE1^amp_hi^ subgroup.

## Author contributions statement

AMYC, EE, CBG and MK conceived the study design. AMYC, EE, JBM, HH, CN, NW, YO, SL, KW, PFR, FW carried out experiments. XG, DGM, PN, NL, LC provided resources. MK and LL analysed the data. AMYC and MK wrote the first draft. All authors were involved in writing the paper and had final approval of the submitted and published versions.

Reference 37 is cited only in the supplementary material.

## Supporting information


**Supplementary materials and methods**
**Figure S1.** *CCNE1* copy number by NanoString and digital PCR
**Figure S2.** IHC of inducible cell lines
**Figure S3.** Correlation between the IHC assay and NanoString/digital PCR
**Figure S4.** Determination of the optimal cut‐off for percentage positive tumour nuclei by IHC to predict *CCNE1* high level amplification by CISH
**Figure S5.** Visual scoring of IHC
**Figure S6.** Prediction of high level *CCNE1* amplification by IHC
**Figure S7.** Visual scoring of *CCNE1* RNA CISH
**Figure S8.** Univariate Kaplan Meier survival analysis
**Table S1.** Agreement between *CCNE1* copy number by NanoString/digital PCR and CISH
**Table S2.** Summary of ROC analysis using visual scoring cut off
**Table S3.** Sensitivity/specificity using 60% cut‐off in the combined cohort
**Table S4.** Clinical characteristics of the two cohorts
**Table S5.** Multivariable analysis of combined *CCNE1* high‐level amplification and protein overexpression status in the combined set
