# Supplementary material for: Combined CCNE1 high‐level amplification and overexpression is associated with unfavourable outcome in tubo‐ovarian high‐grade serous carcinoma
Source: J Pathol Clin Res. 2020 May 11;6(4):252–62. doi: 10.1002/cjp2.168 (PMC7578325; doi:10.1002/cjp2.168)
Supplement: Supplementary file 1 — Supplementary materials and methods Figure S1. CCNE1 copy number by NanoString and digital PCR Figure S2. IHC of inducible cell lines Figure S3. Correlation between the IHC assay and NanoString/digital PCR Figure S4. Determination of the optimal cut‐off for percentage positive tumour nuclei by IHC to predict CCNE1 high level amplification by CISH Figure S5. Visual scoring of IHC Figure S6. Prediction of high level CCNE1 amplification by IHC Figure S7. Visual scoring of CCNE1 RNA CISH Figure S8. Univariate Kaplan Meier survival analysis Table S1. Agreement between CCNE1 copy number by NanoString/digital PCR and CISH Table S2. Summary of ROC analysis using visual scoring cut off Table S3. Sensitivity/specificity using 60% cut‐off in the combined cohort Table S4. Clinical characteristics of the two cohorts Table S5. Multivariable analysis of combined CCNE1 high‐level amplification and protein overexpression status in the combined set [file CJP2-6-252-s001.pdf]

Combined CCNE1 high-level amplification and overexpression is associated with unfavourable outcome in tubo-ovarian high-grade serous carcinoma.

Chan AMY *et al*, *J Pathol Clin Res*, DOI 10.1002/cjp2.168

Contents

Supplementary Materials and Methods

Figure S1. *CCNE1* copy number by NanoString and digital PCR

Figure S2. Immunohistochemistry of inducible cell lines

Figure S3. Correlation between the IHC assay and NanoString/digital PCR

Figure S4. Determination of the optimal cut-off for percentage positive tumour nuclei by immunohistochemistry to predict *CCNE1* high level amplification by CISH

Figure S5. Visual scoring of immunohistochemistry

Figure S6. Prediction of high level *CCNE1* amplification by immunohistochemistry

Figure S7. Visual scoring of *CCNE1* RNA CISH

Figure S8. Univariate Kaplan Meier survival analysis

Table S1. Agreement between *CCNE1* copy number by NanoString/digital PCR and CISH

Table S2. Summary of ROC analysis using visual scoring cut off

Table S3. Sensitivity/specificity using 60% cut-off in the combined cohort

Table S4. Clinical characteristics of the two cohorts

Table S5. Multivariable analysis of combined *CCNE1* high-level amplification and protein overexpression status in the combined set.

Supplementary Materials and Methods

The reference numbers refer to the main text.

CCNE1 DNA CISH

Four µm sections were cut from previously constructed tissue microarrays (TMAs), de-paraffinised, pretreated with proteinase K (3 min) and citrate-based antigen retrieval buffer at 80°C (1 hr), followed by pepsin (45 sec). The pretreated slides were dehydrated and air-dried. DIG-labelled *CCNE1* probe (Empire Genomics, Buffalo, NY, USA) was premixed with hybridisation buffer and heat denatured. Hybridisation was carried out at 37°C for 16-18 hours in ACD HybEZ II over. Multiple washes were performed at 73°C from 2XSSC to 2XSSC/NP40, and then stringent wash following the hybridisation. After the washes, a homemade levamisole solution was used for 15 min incubation to remove endogenous AP activity. A solution of 10% normal sheep serum, 2% BSA, 0.05% Tween-20 was used as blocking solution as well as the following antibody diluent. After 30 min of blocking, AP-conjugated sheep anti-DIG antibody (sheep anti-DIG-AP, Roche) was diluted at 1:800 and applied to the slides and incubated for 2 hours. TBS-T buffer was used for the washes between incubations. Each wash step was performed for 5 min three times. AP substrate (NBT-BCIP tablet) was applied to the slides after the antibody incubation. The color development was closely monitored and the reaction was stopped with KTBT buffer when it reached the desired intensity. Counterstaining was then performed with Fast Red, followed by conventional dehydration and mounting.

RNA CISH

The *CCNE1* RNA probe was purchased from ACDBio (Newark, CA, USA), and RNA CISH assay was performed using the RNAscope detection kit under conditions recommended by the manufacturer (ACDBio, USA) with minor modifications. Four µm sections were cut from TMA blocks. The sections were de-paraffinised, washed with 60°C water for 5 min, and then digested with proteinase K (DAKP S3020, RTU) for 3 minutes. Afterwards, the slides were treated with 3% H2O2 at room temperature for 10 min, dehydrated and air dried thoroughly. The RNA probe was then applied to the slides and the incubation was done at 42°C overnight, followed by 2 times washing with RNAscope wash buffer (ACDBio, Newark, CA, USA). For detection of the *CCNE1* RNA expression levels, eight detection reagents from the kit were added to the slides stepwise, each with 2 times wash with wash buffer. At the end, the signal was monitored under microscope for a desired intensity.

NanoString

H&E-stained slides were marked for regions of high tumour cellularity. Corresponding formalin-fixed paraffin-embedded (FFPE) tissue blocks were cored with two 1-mm biopsy punches. DNA was then extracted by use of the QIAamp DNA Micro Kit protocol (Qiagen, Hilden, Germany). DNA was quantified with the Qubit dsDNA HS Assay Kit and Qubit 2.0 fluorometer (ThermoFisher Scientific, Waltham, MA, USA). Gene copy number variations were assessed on the NanoString nCounter platform (Seattle, WA, USA) with the nCounter Cancer CN Assay (Seattle, WA, USA) for 90 target genes including *CCNE1*. Four hundred nanograms of genomic DNA was digested by Alu1, according to the manufacturer's protocol, and hybridised with reporter and capture probe sets for 18 h at 65°C. Samples were processed, and signals were counted post-hybridisation on the nCounter Prep Station and Digital Analyzer. Probe counts were normalised to invariant controls by the use of NSOLVER ANALYSIS v2.0. Normal fallopian tube and lymph node tissue served as reference controls.

Digital PCR

CCNE1 copy number was assessed by digital PCR using the QuantStudio 3D Digital PCR platform (ThermoFisher Scientific, Waltham, MA USA). Tumour DNA was diluted to bring the concentration of *CCNE1* target molecules to 200-2000 copies/µL. The PCR reaction for each sample consisted of 7.5 µL 2X 3D Master Mix, 0.75 µL FAM-labelled *CCNE1* TaqMan copy number assay (Hs07137484_cn), 0.75 µL VIC labelled *RNaseP* TaqMan copy number reference assay and diluted DNA up to 15 µL volume. Each sample was loaded onto a 3D digital PCR 20K chip and run on a ProFlex™ 2xFlat PCR thermocycler following the manufacturer's protocol. Digital imaging was performed on the QuantStudio™ 3D Digital PCR Instrument and results were analysed using the QuantStudio™ 3D Analysis Suite Software version 3.0 (Applied Biosystems by ThermoFisher Scientific). *CCNE1* copy number was determined by dividing the number of *CCNE1* copies/µL by *RNaseP* copies/µL and multiplying the ratio by 2 and then normalised to average *CCNE1* copy number from normal fallopian tube and lymph node tissues for consistency in comparison with NanoString copy number data.

Inducible cell line control

pEF1a-rtTA-IRES-GFP from a Tet-ON inducible lentiviral vector system (Takara Bio Group, Mountain View, California, USA) was packaged into lentiviral particles by co-transfecting HEK293FT cells (Thermofisher, Waltham, MA) with psPAX2 and pMD2.G (gifts from Didier Trono (Addgene plasmid numbers 12260 and 12259, respectively)). Viral particles were concentrated from cell culture supernatant and underlaid with 2 mL 20% sucrose in PBS by ultracentrifugation at 50,000 x g for 2 h. The titres were determined by qPCR lentivirus titration kit and were generally ~108 IU/mL (Applied

Biological Materials, Richmond, BC). The pEF1a-rtTA-IRES-GFP will simultaneously produce tetracycline activator and GFP transcription bicistronically.

Reference TMA establishment

A range of CCNE1 expression cell lines were created and embedded into HistoGel using the method described previously [30]. Briefly, these cells were seeded at 5×10^6 cells per T75 (25 ml of media). The next day cells were treated with increasing amount of Doxycycline (Santa Cruz Biotechnology, Dallas, Texas, USA) for 24 hours to generate cells with increasing amounts of CCNE1. K562 cells are suspension cells, therefore cells expressing different levels of CCNE1 can be harvested at the same time by centrifugation. Ten percent of the cells were lysed for Western blotting and 90% were embedded in HistoGel. Cells were washed, resuspended and fixed in 10% formalin (Thermo Fisher Scientific, Burlington, ON, Canada) by incubating on ice for 60 min. During this time, cells were counted using a Moxi Z cell counter. Fixed cells were washed and dried cell pellets were resuspended in 65°C molten HistoGel (Thermo Fisher, Canada) at approximately $2x10^7$ cells/100µL of Histogel. The gel-embedded cells were solidified at 4°C and overlaid with 70% ethanol until processing into paraffin-embedded blocks. Formalin-fixed paraffin embedded cell blocks were then constructed into tissue microarray.

Immunohistochemistry

Commercially available CCNE1 antibodies

Antibody	Clone	Species	Vendor	Cat#	Assay condition	Comment
CCNE1 (1)	13A3	MM	Leica	NCL-CYCLIN E	ER1(30), 1:50	Same as (4)
CCNE1 (2)	EP435E	RM	Abcam	Ab33911	TRS high and low, 1:200	Non-specific cytoplasmic staining
CCNE1 (3)	-	RP		ab74276	ER2(30), 1:100	Poor signal/noise ratio
CCNE1 (4)	13A3	MM	Abcam	Ab9517	ER2(30)+1hr, 1:100	Good stain but lacks specificity, also recognises CCNE2; discontinued
CCNE1 (5)	EP126	RM	Cell Marque	CMQ-AC0120RUOC	TRS high, 1:600	Best staining, specific antibody
CCNE1 (6)	HE12	MM	Abcam	ab3927	TRS high, 1:800	Good staining, specific antibody

Visual scoring

Distribution % was assessed in 10-20% tier categories blinded to outcome data. An example image library.

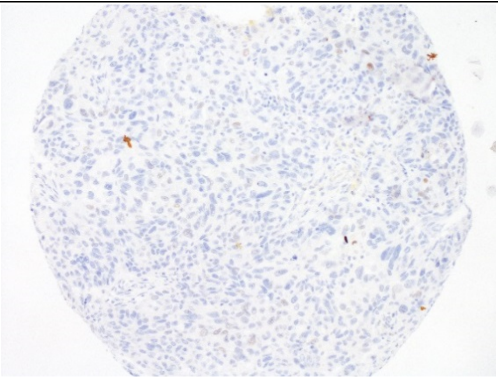<br>**<20%**	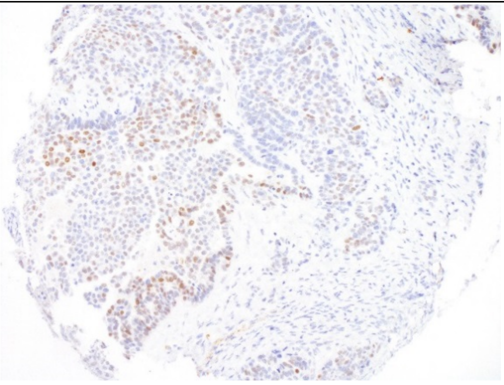<br>**20-39%**
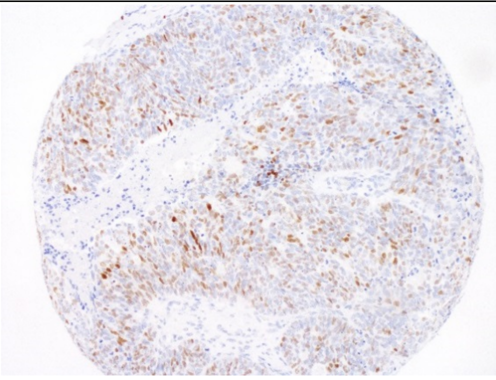<br>**40-49%**	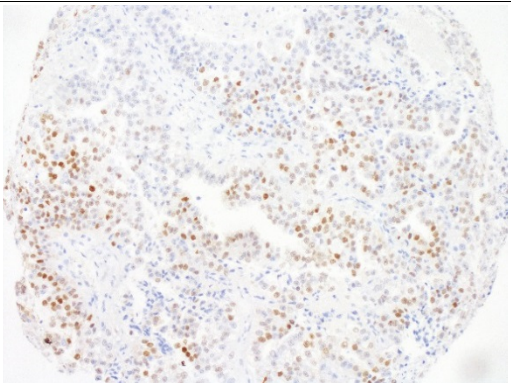<br>**50-59%**
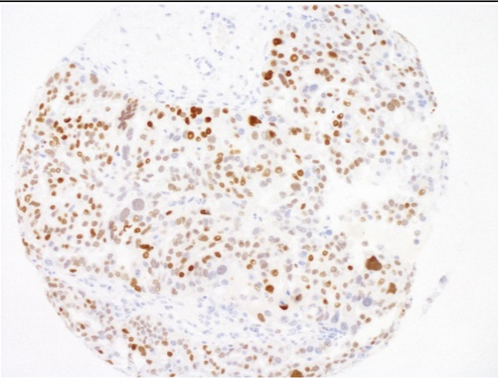<br>**60-79%**	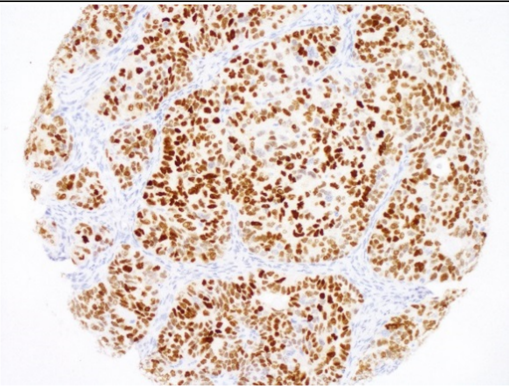<br>**80-100%**

Adherence to REMARK guidelines

**The REMARK checklist**	**RESPONSE**
**INTRODUCTION**<br>1. State the marker examined, the study objectives, and any pre-specified hypotheses.	1. M**arker**: CCNE1<br>**Objectives** “We aimed to investigate whether immunohistochemistry (IHC) can be used to identify CCNE1 amplification status and to validate whether CCNE1 high-level amplification and overexpression are prognostic in HGSC using in large training and a testing sets” abnormalities of the G1/S cell cycle checkpoint complex.”
**MATERIALS AND METHODS**<br>**Patients**<br>2. Describe the characteristics (for example, disease stage or co-morbidities) of the study patients, including their source and inclusion and exclusion criteria.<br>3. Describe treatments received and how chosen (for example, randomised or rule-based).<br>Specimen characteristics<br>4. Describe type of biological material used (including control samples) and methods of preservation and storage.	2. Case selection/ inclusion: High-grade serous carcinoma identified from 2 studies. Histotype diagnosis confirmed by a combination of WT1/p53 immunohistochemistry<br>3. Treatment: patients recruited through an era of homogeneous platinum-based adjuvant chemotherapy, which the majority received Table 2. Multivariate analysis were adjusted for treatment.<br>4. Biological material: tissue microarrays from formalin fixed paraffin embedded tissue. Controls for DNA content derived from NanoString and digital PCR, for immunohistochemistry: high expressor control: clear cell carcinoma, low expressor control: placenta, negative control: appendix mucosa.

**Assay methods**<br>5. Specify the assay method used and provide (or reference) a detailed protocol, including specific reagents or kits used, quality control procedures, reproducibility assessments, quantitation methods, and scoring and reporting protocols. Specify whether and<br>how assays were performed blinded to the study endpoint.	5. Assays described in detail in methods and supplementary methods: CISH, IHC, Nanostring, digital PCR, inducible cell line controls, image analysis, visual scoring
**Study design**<br>6. State the method of case selection, including whether prospective or retrospective and whether stratification or matching (for<br>example, by stage of disease or age) was used.<br>7. Precisely define all clinical endpoints examined.<br>8. List all candidate variables initially examined or considered for inclusion in models.<br>9. Give rationale for sample size; if the study was designed to detect a specified effect size, give the target power and effect size.	6. Retrospective case selection from 2 prior studies: one population based incident cases from one Canadian province, the second a collection biobank cases across Canada. Duplicate cases were removed<br>7. The end point is ovarian cancer specific (ie. death due to ovarian cancer censoring death of other causes) death<br>8. Age, Stage, cohort, residual disease after surgery, treatment<br>9. The five year survival is approximately 40% and follow up exceeds 5 years for the majority of cases. Assuming the prevalence of the amplification is 20%, with 60% death, the training set will have >80% power to detect a HR of 1.4 of survival at a type I error rate of 0.05, with the smaller group having the poorer prognosis.
**Statistical analysis methods**<br>10. Specify all statistical methods, including details of any variable selection procedures and other model-building issues, how model	10. Stated in methods. Missing data were separately fitted into model as missing data. Cox proportional hazards assumption was verified by visual inspection of Kaplan-Meier curves.

assumptions were verified, and how missing data were handled.<br>11. Clarify how marker values were handled in the analyses; if relevant, describe methods used for cut point determination.	11. Cut-off determination performed by ROC analysis
**RESULTS**<br>**Data**<br>12. Describe the flow of patients through the study, including the number of patients included in each stage of the analysis (a diagram<br>may be helpful) and reasons for dropout. Specifically, both overall and for each subgroup extensively examined report the number of patients and the number of events.<br>13. Report distributions of basic demographic characteristics (at least age and sex), standard (disease-specific) prognostic variables, and tumour marker, including numbers of missing values.	12. Described in Results section as optimisation cohort, training and testing set and combined analysis<br>13. Reported in Table 1

**Analysis and presentation**<br>14. Show the relation of the marker to standard prognostic variables.<br>15. Present univariable analyses showing the relation between the marker and outcome, with the estimated effect (for example, hazard ratio and survival probability). Preferably provide similar analyses for all other variables being analysed. For the effect of a tumour marker on a time-to-event outcome, a Kaplan-Meier plot is recommended.<br>16. For key multivariable analyses, report estimated effects (for example, hazard ratio) with confidence intervals for the marker and, at least for the final model, all other variables in the model.<br>17. Among reported results, provide estimated effects with confidence intervals from an analysis in which the marker and standard prognostic variables are included, regardless of their statistical significance.<br>18. If done, report results of further investigations, such as checking assumptions, sensitivity analyses, and internal validation.	14. Reported in Table 4<br>15. Supplementary Table S5<br>16. Reported in Table 3<br>17. NA<br>18. Model assumption were assessed by visual inspection of Kaplan Meier curves
**DISCUSSION**<br>19. Interpret the results in the context of the pre-specified hypotheses and other relevant studies; include a discussion of limitations of the study.<br>20. Discuss implications for future research and clinical value.	19. See discussion<br>20. See discussion
[37] Altman DG, McShane LM, Sauerbrei W *et al*. Reporting Recommendations for Tumor Marker Prognostic Studies (REMARK): Explanation and Elaboration. *PLoS Med* 2012; **9:** e1001216. https://doi.org/10.1371/journal.pmed.1001216	

Supplementary Figures

Figure S1. *CCNE1* copy number by NanoString and digital PCR

A separate optimization cohort was sourced from local cases (48 HGSC). NanoString copy number assay was performed. The median copy number was 2.58 (range 1.4-19.3). Cases were classified as LOH with copy numbers < 1.5, diploid 1.5 - 2.4, gain 2.4-5.0, amplified 5.0-8.0, and high-level amplification > 8.0. Four cases showed high-level amplifications (three cases >17 copies; one case > 8 copies). High-level amplified cases were confirmed by digital PCR and raw copy number values correlated well between NanoString and digital PCR assay (Spearmen correlation of 0.821). There was one case with 5.63 copies by NanoString but 8.93 copies by digital PCR, which changed the category to high-level amplification based on digital PCR. Using 8 copies by at least one assay as cut-off, 5/48 (10.4%) of cases from the optimisation cohort showed high-level amplification.

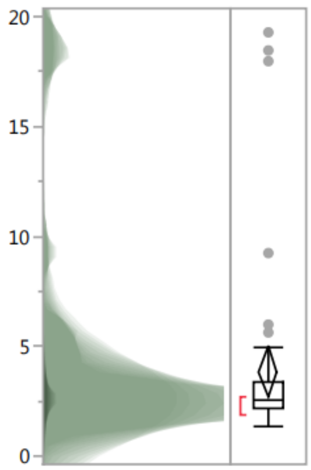

Figure S2. Immunohistochemistry of inducible cell lines

Representative cell block IHC images and digital mark up images of the two optimised assays. The digital image analysis was guided by trained pathologist to bin intensity as negative (blue), weak (yellow), moderate (orange) and high (red).

A. EP126

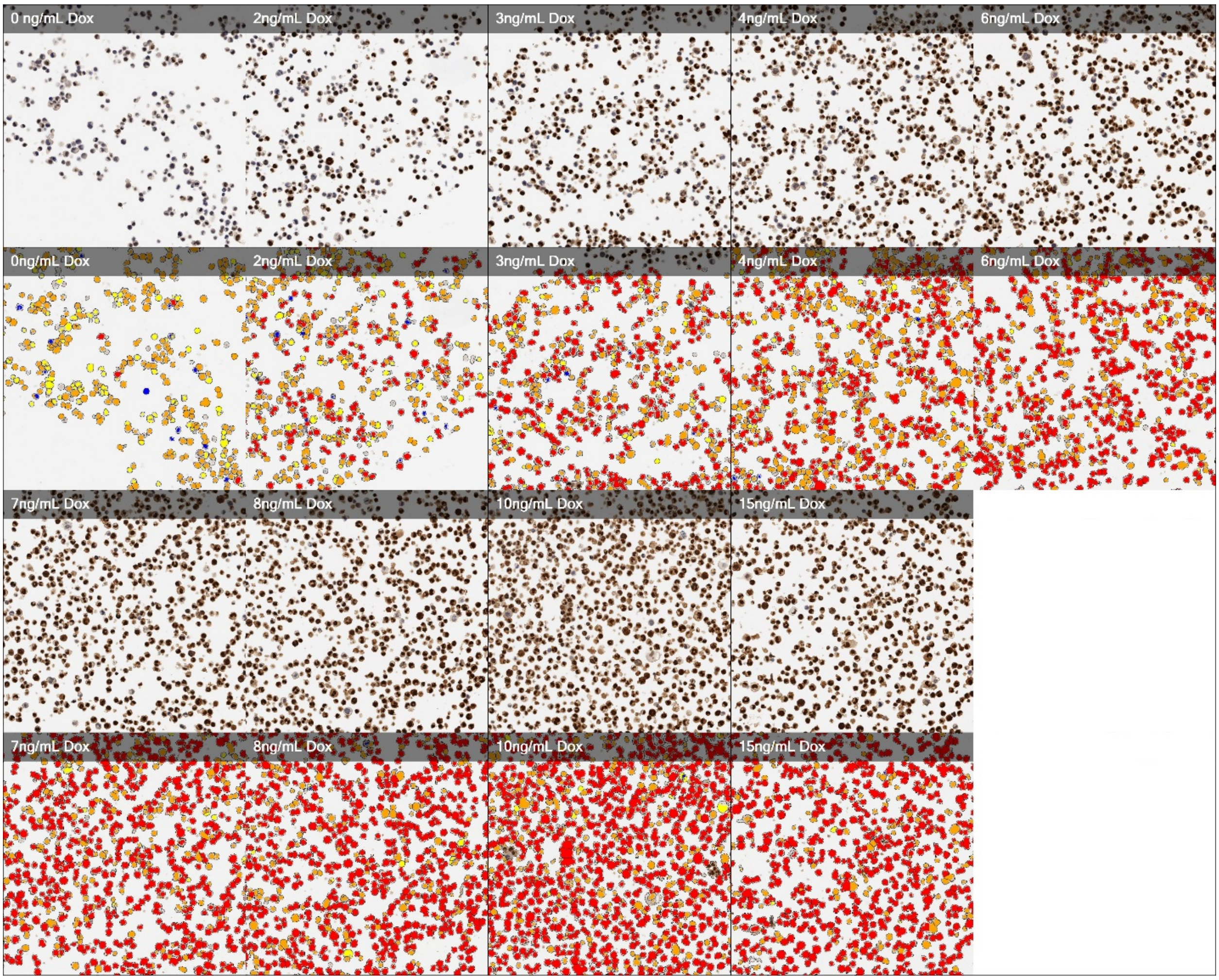

B. HE12

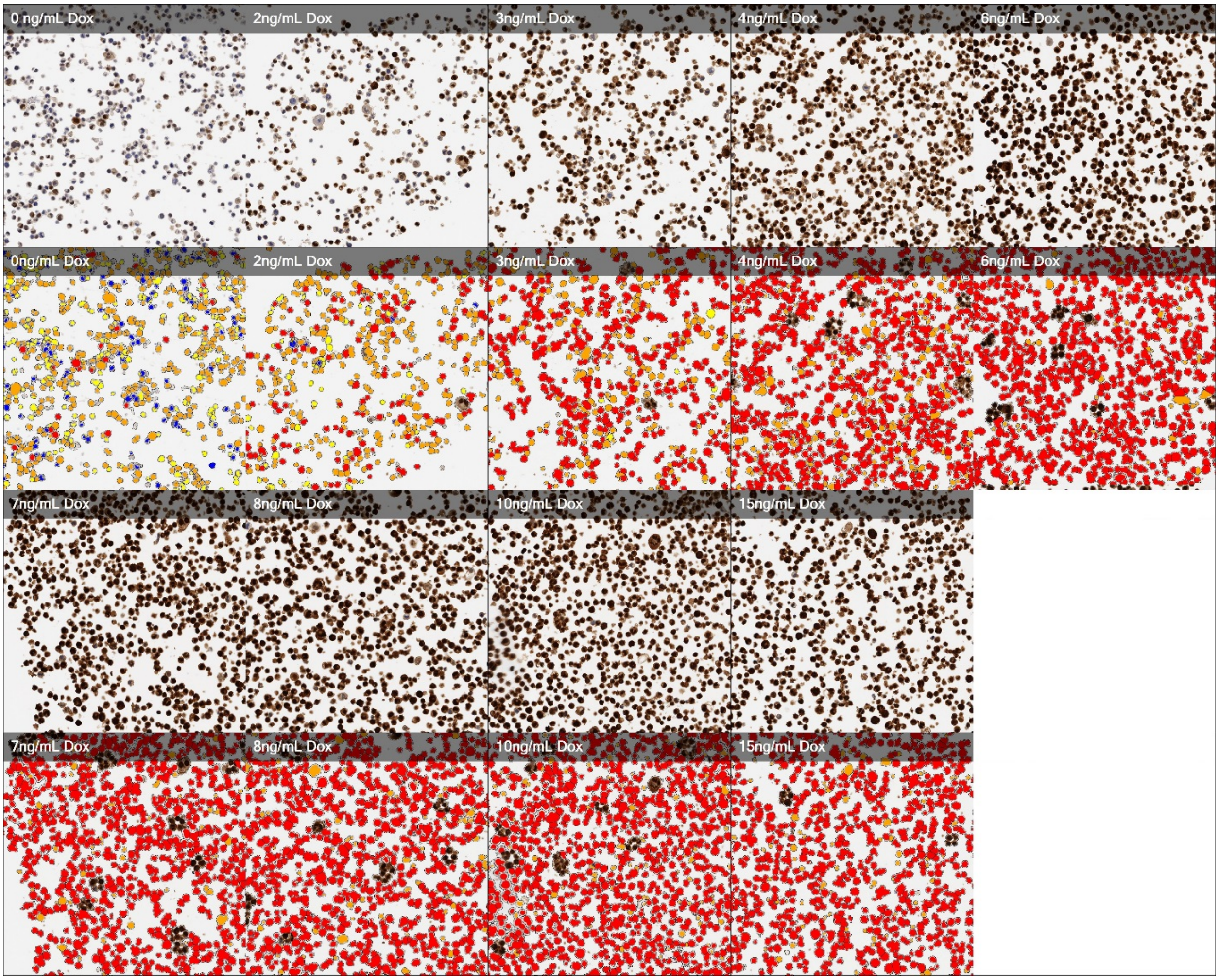

0 ng/mL Dox
2ng/mL Dox
3ng/mL Dox
4ng/mL Dox
6ng/mL Dox
0ng/mL Dox
2ng/mL Dox
3ng/mL Dox
4ng/mL Dox
6ng/mL Dox
7ng/mL Dox
8ng/mL Dox
10ng/mL Dox
15ng/mL Dox
7ng/mL Dox
8ng/mL Dox
10ng/mL Dox
15ng/mL Dox

Figure S3. Correlation between the IHC assay and NanoString/digital PCR

Whether the NanoString/digital PCR copy number data could be used to optimise the CCNE1 immunohistochemistry assay, including cut-off determination, was explored. We examined the correlation between the optical density from IHC assay and copy number from NanoString/digital PCR. Spearman's correlation coefficient ranged between 0.35 (EP126) and 0.49 (HE12). This did not allow us to discriminate between the 2 IHC assays. Next we tried to establish a cut-off for IHC that might predict high level amplification.

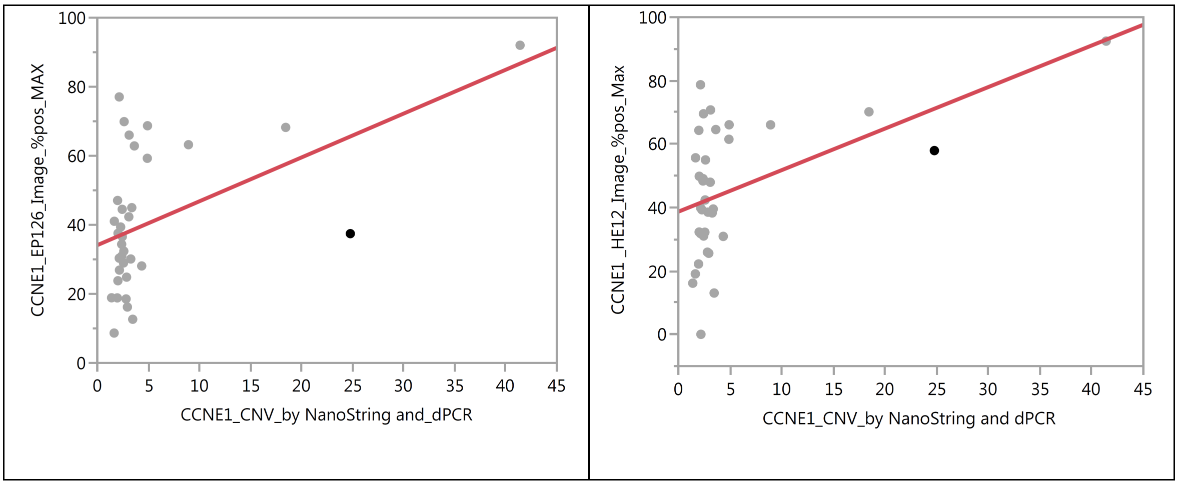

A cut-off of 60% positive tumour cells had sensitivity of 75% for both IHC assays and a specificity of 38% and 30% for antibody EP126 and HE12, respectively. A cut-off of 40% increased the sensitivity to 100% but decreased specificity to approximately 20%. Based on this, we preliminarily concluded that a cut-off between 40% and 60% would have sufficient sensitivity to detect almost all high level amplified cases.

Figure S4. Determination of the optimal cut-off for percentage positive tumour nuclei by IHC to predict *CCNE1* high level amplification by CISH.

The area under the curve (AUC ROC) for immunohistochemistry was 0.787 suggesting modest accuracy in predicting high level amplification status. ROC analysis revealed 61% as the optimal cut-off to balance sensitivity/specificity to identify high-level amplifications.

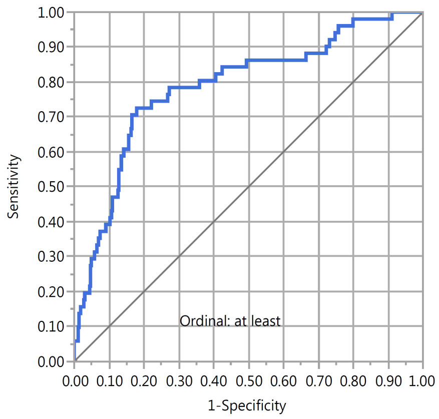

Figure S5. Visual scoring of immunohistochemistry

We decided on 20%-increment visual scoring creating a 6-tier scoring system (see supplementary methods):

1 - <20%

2 - 20 - 39%

3 - 40 - 49 %

4 - 50 - 59%

5 - 60 – 79%

6 - 80% -100%.

There was strong correlation between continuous image analysis scores and visual categories Spearmen r=0.896. Using a 60% cut off, the agreement between image analysis and visual scoring achieved a Cohen's kappa of 0.726.

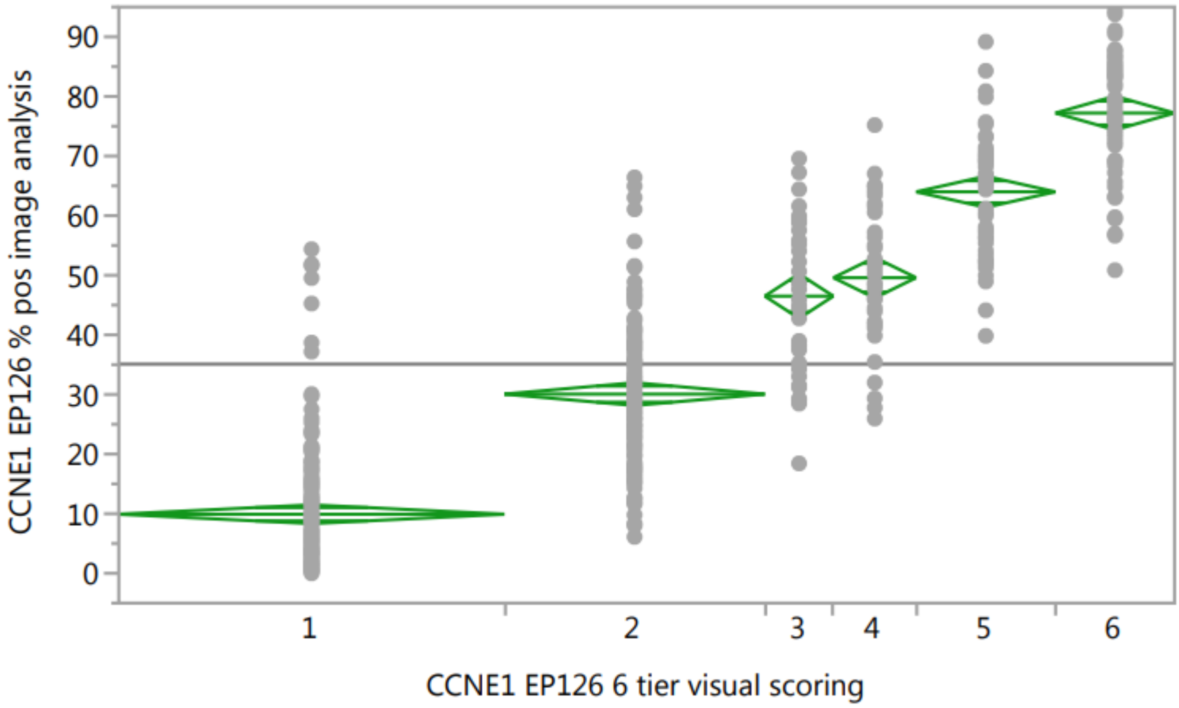

Figure S6. Prediction of high level *CCNE1* amplification by immunohistochemistry

We next tested the ability of visual scoring categories to predict *CCNE1* high level amplification by CISH. The area under the curve was slightly better than the image analysis data (AUC=0.825). Again, the sensitivity started to plateau at about 75%.

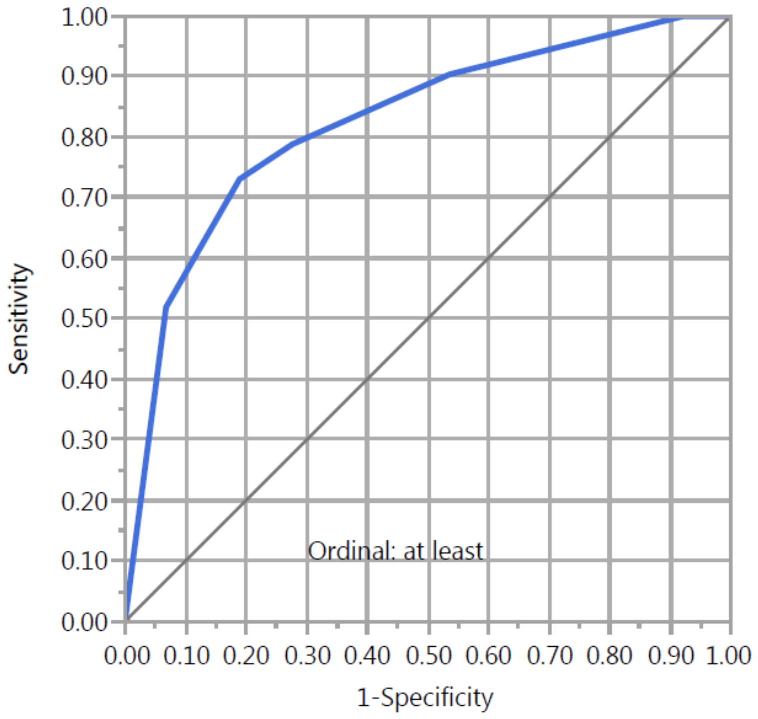

Figure S7. Visual scoring of *CCNE1* RNA CISH

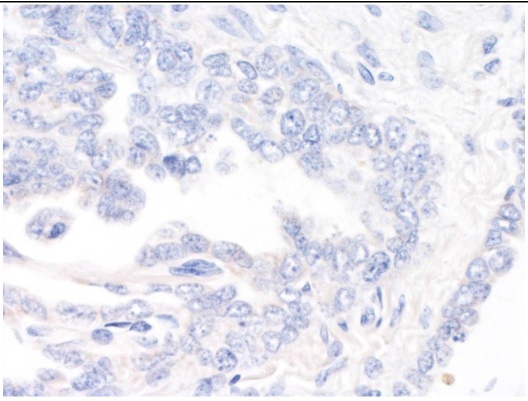

Absent

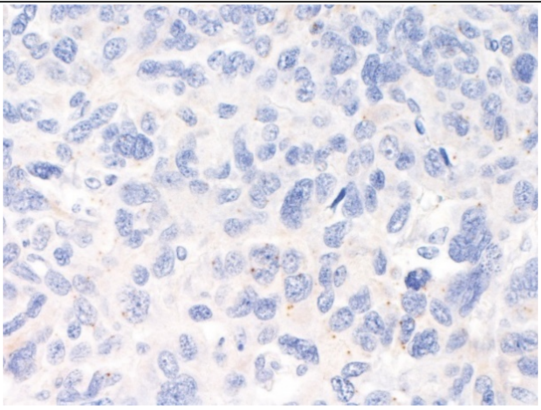

Weak

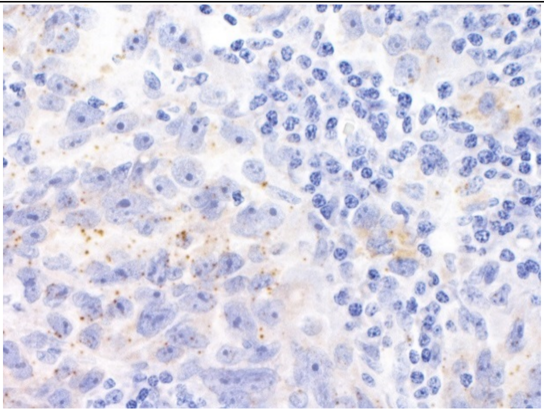

Moderate

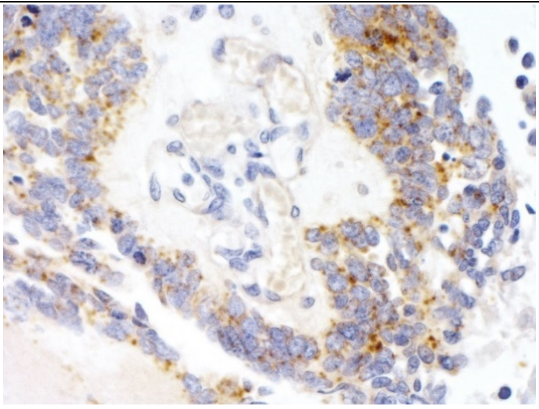

Strong

Figure S8. Univariate Kaplan Meier survival analysis

	Testing Set							Validation Set						
DNA CISH<br>Number at risk:	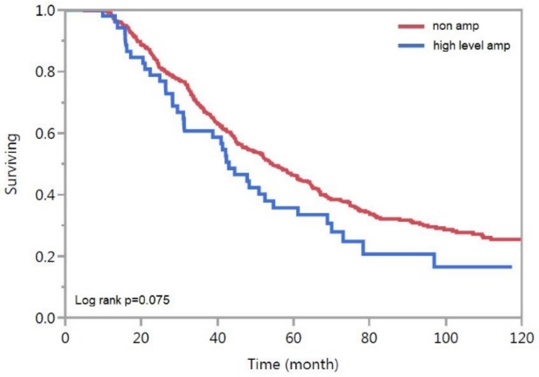							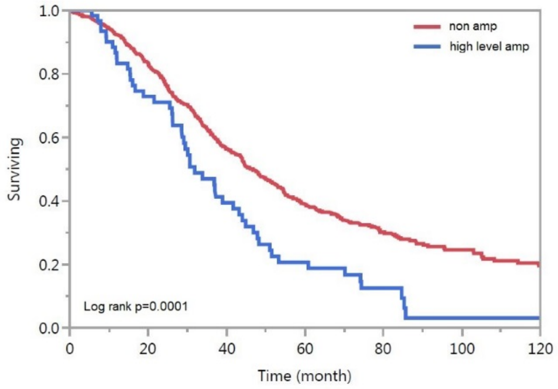						
Non amp	412	365	251	166	96	63	38	588	457	291	161	89	48	26
HLA	52	30	29	17	6	4	1	62	41	22	12	5	2	1
RNA CISH<br>Number at risk:	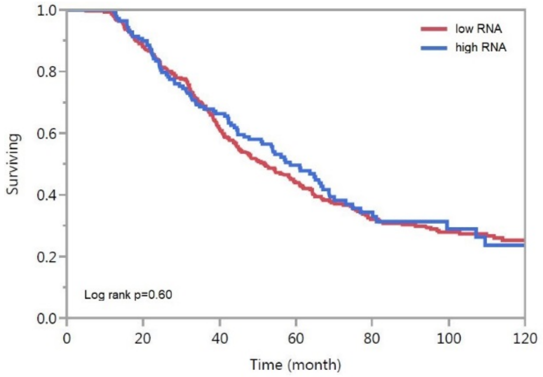							NA						
Low	303	264	179	121	76	56	31							
High	140	126	90	55	25	12	8							
IHC<br>Number at risk:	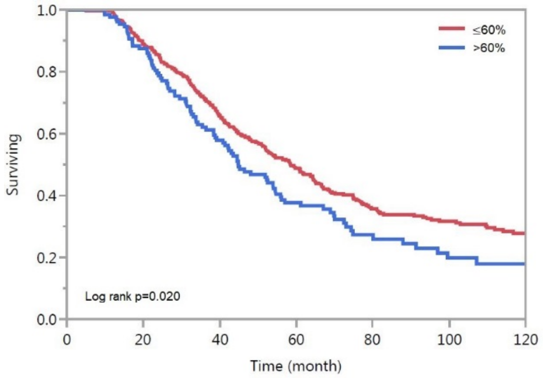							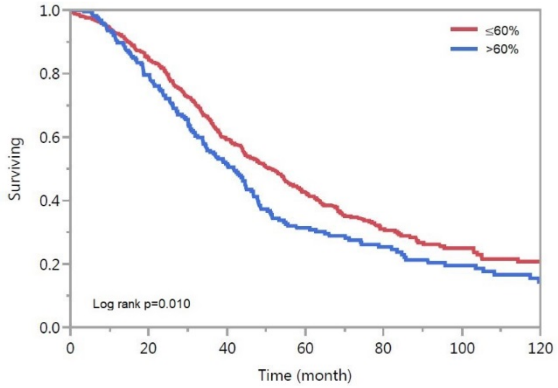						
≤60	399	351	254	174	101	69	41	537	431	285	162	79	41	24
>60	129	112	69	40	20	13	8	227	163	98	52	34	22	13

Supplementary Tables

Table S1. Agreement between *CCNE1* copy number by NanoString/digital PCR and CISH

		***CCNE1* NanoString/digital PCR**				
		LOH	Diploid	Gain	Amplification	High-level amplification
***CCNE1* DNA CISH**	Diploid	1	9	12	0	0
	Low level amplification	0	3	1	0	0
	High level amplification	0	0	0	0	4

All 4 cases available data for both showing high level amplification by NanoString/digital PCR and by CISH. However, the discrimination between diploid and amplification was not good. Therefore, *CCNE1* CISH data in main analyses were categorised as high-level amplification versus no high-level amplification.

Table S2. Summary of ROC analysis using visual scoring cut off

Testing set

Visual scoring cut-off	Sensitivity	Specificity	Accuracy	AUC ROC
≥40%	78.9%	64.6%	66%	0.717
≥50%	78.9%	72.3%	73%	0.756
≥60%	73.1%	81.1%	80%	**0.771**
≥80%	51.9%	93.2%	88%	0.726

Validation set

Visual scoring cut-off	Sensitivity	Specificity	Accuracy	AUC ROC
≥40%	96.8%	58.3%	62%	0.776
≥50%	93.6%	67.4%	70%	0.805
≥60%	88.7%	74.8%	76%	**0.812**
≥80%	53.2%	93.9%	90%	0.736

Combined cohort

Visual scoring cut-off	Sensitivity	Specificity	Accuracy	AUC ROC
≥40%	88.6%	60.4%	63.2%	0.745
≥50%	86.8%	67.2%	69.2%	0.770
≥60%	**81.6%**	77.4%	77.7%	**0.794**
≥80%	52.6%	93.3%	89.2%	0.729

Table S3. Sensitivity/specificity using 60% cut-off in the combined cohort

	$CCNE1^{amp}$	$CCNE1^{nonamp}$	Total
$CCNE1^{hi}$	93 (81.6%)	226 (22.6%)	319
$CCNE1^{lo}$	21 (18.4%)	774 (77.4%)	795
Total	114	1000	1114

$CCNE1^{amp}$ - *CCNE1* high-level amplification (>8 copies by CISH)
$CCNE1^{nonamp}$ – negative for *CCNE1* high-level amplification (≤8 copies by CISH)
$CCNE1^{hi}$ – CCNE1 protein overexpression by IHC with ≥60% positive tumour cells and at least 5% strongly staining cells
$CCNE1^{lo}$ – negative for CCNE1 protein overexpression by IHC with <60% positive tumour cells or <5% strongly staining cells

Table S4. Clinical characteristics of the two cohorts

**Variable**	**Testing set**	**Validation set**
Cohort Name	OVAL BC	COEUR
N (HGSC)	528	764
Age, mean (SD)	61.0 (10.0)	61.8 (11.0)
Stage; *N* (%)		
Stage I	40 (7.6%)	51 (6.7%)
Stage II	56 (10.6%)	86 (11.3%)
Stage III	356 (67.4%)	526 (68.5%)
Stage IV	56 (10.6%)	71 (9.3%)
Unknown	20 (3.8%)	3 (3.9%)
Surgical outcome; *N* (%)		
Complete resection	187 (35.4%)	144 (18.8%)
Optimal resection	135 (25.6%)	210 (27.5%)
Suboptimal resection	181 (34.3%)	216 (28.3%)
Unknown	25 (4.7%)	194 (25.4%)
Platinum-based chemotherapy *N* (%)		
None	6 (1.1%)	11 (1.4%)
Neoadjuvant	98 (18.6%)	36 (4.7%)
Adjuvant	417 (79.1%)	672 (87.9%)
Unknown	6 (1.1%)	45 (5.9%)
Median follow up time for censored cases; months (SD)	81.3 (39.0)	58.8 (36.6)

Table S5. Subgroup comparison: multivariable analysis of combined *CCNE1* high-level amplification and protein overexpression status in the combined set.

	**Reference**	**$CCNE1^{nonamp\_lo}$**	**$CCNE1^{nonamp\_hi}$**	**$CCNE1^{amp\_lo}$**
Comparator	*N* (%) *N*=1114	HR (95% CI, *p*)	HR (95% CI, *p*)	HR (95% CI, *p*)
$CCNE1^{amp\_hi}$	93 (8.3%)	1.84 (1.42-2.35, p<0.0001)	1.55 (1.17-2.05, p=0.0028)	2.29 (1.32-4.28, p=0.0026)
$CCNE1^{amp\_lo}$	21 (1.9%)	0.80 (0.44-1.33, p=0.80)	0.68 (0.37-1.14, p=0.15)	X
$CCNE1^{nonamp\_hi}$	226 (20.3%)	1.18 (0.98-1.43, p=0.076)	X	X
$CCNE1^{nonamp\_lo}$	774 (69.5%)	X	X	X

*multivariable analysis adjusted for set, age, stage, surgical outcome and platinum-based chemotherapy
